# Supplementary material for: Predicting visual function by interpreting a neuronal wiring diagram
Source: Nature. 2024 Oct 2;634(8032):113–23. doi: 10.1038/s41586-024-07953-5 (PMC11446822; doi:10.1038/s41586-024-07953-5)
Supplement: Supplementary file 6 — CRF and ERF predictions for individual TmY4 and TmY9 cells. Analogous to Supplementary Data 3, but for TmY target types. Shown are the top four monosynaptic pathways, the strongest pathway passing through each of the top ten intermediary types (ranking from Extended Data Fig. 7), and the trisynaptic pathway Tm1–TmY–Dm3–TmY (see the section entitled Prediction of spatial normalization). [file 41586_2024_7953_MOESM6_ESM.zip › DataS4/TmY9q/720575940608966613.pdf]

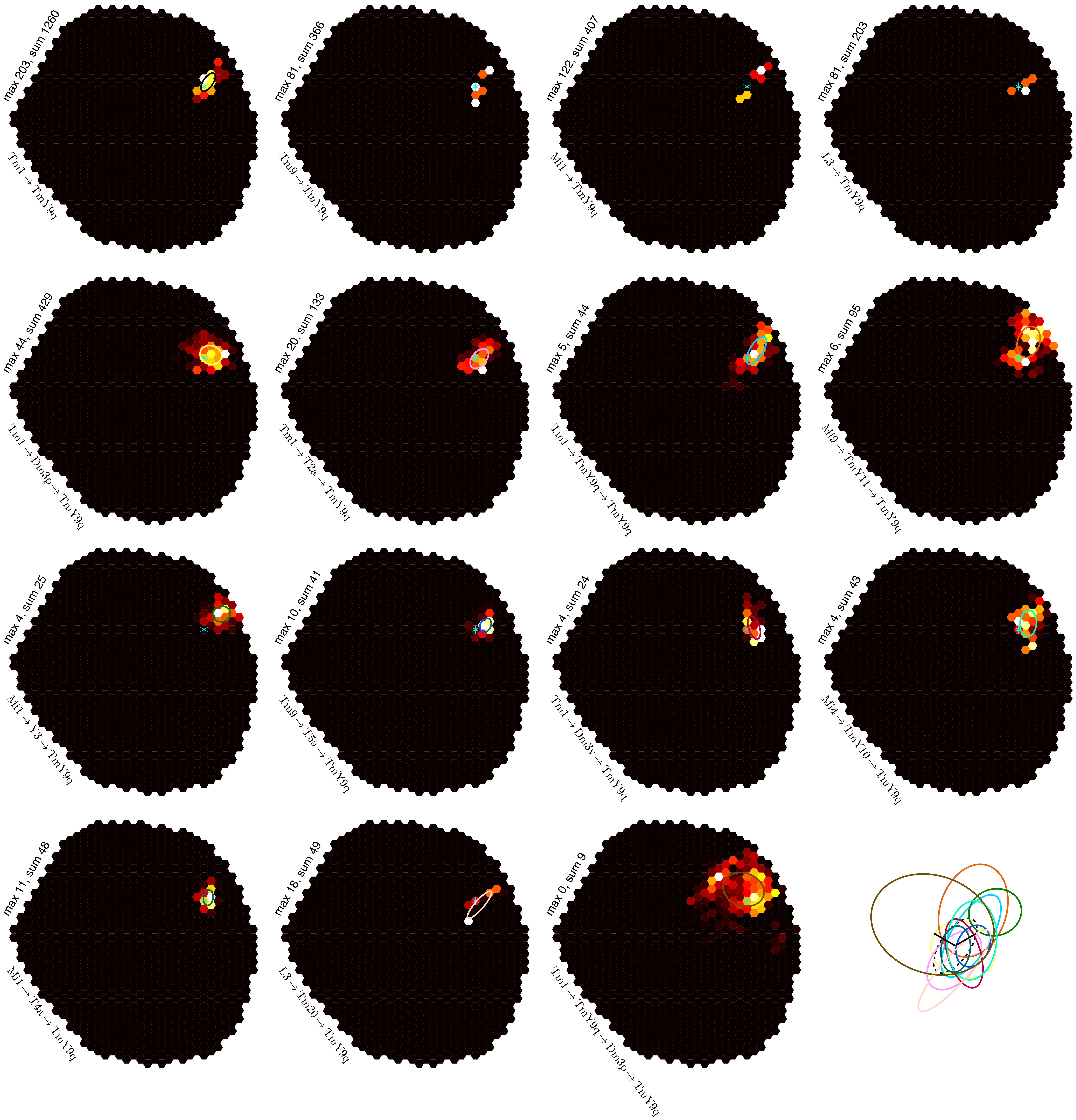
max 203, sum 1260
Tm1 → TmY9q
max 81, sum 366
Tm9 → TmY9q
max 122, sum 407
Mi1 → TmY9q
max 81, sum 203
L3 → TmY9q
max 44, sum 429
Tm1 → Dm3p → TmY9q
max 20, sum 133
Tm1 → T2a → TmY9q
max 5, sum 44
Tm1 → TmY9q → TmY9q
max 6, sum 95
Mi9 → TmY11 → TmY9q
max 4, sum 25
Mi1 → Y3 → TmY9q
max 10, sum 41
Tm9 → T5a → TmY9q
max 4, sum 24
Tm1 → Dm3v → TmY9q
max 4, sum 43
Mi4 → TmY10 → TmY9q
max 11, sum 48
Mi1 → T4a → TmY9q
max 18, sum 49
L3 → Tm20 → TmY9q
max 0, sum 9
Tm1 → TmY9q → Dm3p → TmY9q
